# Preparation, Cytotoxicity, and In Vitro Bioimaging of Water Soluble and Highly Fluorescent Palladium Nanoclusters

**DOI:** 10.3390/bioengineering7010020

**Published:** 2020-02-21

**Authors:** Suresh Thangudu, Poliraju Kalluru, Raviraj Vankayala

**Affiliations:** 1 Department of Chemistry, National Tsing Hua University, Hsinchu 30013, Taiwan; suresh120689@gmail.com; 2 Department of Chemistry, University of Calgary, Calgary, AB T2N 1N4, Canada; raju.poli@gmail.com; 3 Department of Bioscience and Bioengineering, Indian Institute of Technology Jodhpur, Jodhpur, Rajasthan 342037, India

**Keywords:** palladium, nanoclusters, fluorescence, cancer cells, passive targeting, bioimaging

## Abstract

Fluorescent probes offer great potential to identify and treat surgical tumors by clinicians. To this end, several molecular probes were examined as in vitro and in vivo bioimaging probes. However, due to their ultra-low extinction coefficients as well as photobleaching problems, conventional molecular probes limit its practical utility. To address the above mentioned challenges, metal nanoclusters (MNCs) can serve as an excellent alternative with many unique features such as higher molar extinction coefficients/light absorbing capabilities, good photostability and appreciable fluorescence quantum yields. Herein, we reported a green synthesis of water soluble palladium nanoclusters (Pd NCs) and characterized them by using various spectroscopic and microscopic characterization techniques. These nanoclusters showed excellent photophysical properties with the characteristic emission peak centered at 500 nm under 420 nm photoexcitation wavelength. In vitro cytotoxicity studies in human cervical cancer cells (HeLa) cells reveal that Pd NCs exhibited good biocompatibility with an IC_50_ value of >100 µg/mL and also showed excellent co-localization and distribution throughout the cytoplasm region with a significant fraction translocating into cell nucleus. We foresee that Pd NCs will carry huge potential to serve as a new generation bioimaging nanoprobe owing to its smaller size, minimal cytotoxicity, nucleus translocation capability and good cell labelling properties.

## 1. Introduction

Cancer is one of the leading life threatening disease to human life in the world [1]. Clinical management of cancer is currently based on cytoreductive surgery [2]. The degree of cytoreductive surgery is one of the most important factors for prognosis, with improved survival associated with completeness of resection of all visible cancer [3]. However, the current surgical standard is to remove implants larger than 1 cm. Even these lesions are difficult to detect by imaging or visually under open abdomen. Even at this standard, surgically removing abdominal metastases in patients with advanced disease improves survival, and by decreasing the initial tumor burden, efficacy of chemotherapy is enhanced [4,5]. Currently, the gold standard in clinical treatments of cancer is the invasive surgical removal of tumors. The importance of complete resection is emphasized by the success of therapy with the early stage disease. Therefore, it leaves a grand challenge for the development of new imaging modalities that can identify small tumor nodules (<1 mm diameter) will have tremendous clinical significance by enabling image-guided identification for resection of all tumors to ultimately eliminate recurrence and improve treatment success and patient survival [6]. To this end, fluorescent small molecular probes such as cyanine dyes and fluorescein dyes opens a new path to visualize the cancer cell [7]. Even though having its own merits, owing to its lower fluorescence quantum yields and photo bleaching of molecular probes makes it far for practical usage. Thus, many pioneers were started to investigate the alternative and efficient strategies to overcome the previous limitations. After several efforts, fluorescent quantum dots (QDs) [8], metal nanoclusters (MNCs) [9], and metal nanoparticles (MNPs) [10] stand out as alternatives to the conventional molecular probes owing to its unique physiochemical properties such as tunable optical properties, higher fluorescent quantum yields and longtime photostabilities.

Briefly, QDs are inorganic nanocrystals which consists of a semiconductor core and shell with high fluorescence quantum yields and good photostabilities. Besides their ultra-small sizes, tunable photoluminescence properties even in the near infrared (NIR) wavelengths, larger stokes shift of QDs made them as potential alternatives to conventional fluorophores and successfully applied for the detection of cancer cells and drug delivery platforms [11,12]. However, there are several limitations which can severely restrict their utilities in biomedical clinical applications such as, (i) after synthesis of QDs and solubilization remains challenging; (ii) clinical approval of QDs is still questionable; (iii) toxicities of heavy metals such as cadmium, tellurium, selenium etc.; (iv) non-biodegradability and accumulate in the body for prolonged periods, so long term effects are still questionable. On the other hand, MNCs are highly attractive with advantages such as ultra-small sizes, tunable fluorescence, large stokes shift, quantum charging, magnetism, molecular chirality, lower toxicities, etc. As compared to metal nanoparticles, metal nanoclusters (NCs) have attracted special attention due to their unique features and molecule-like properties. Metal NCs usually consist of a few to a hundred atoms, and the sizes are comparable to the Fermi wavelength of electrons [13], which endows them to play an important role in the missing link between single metal atoms and plasmonic metal nanoparticles. In this size regime, the continuous density of states (DOS) breaks up into discrete energy levels. Due to the electrons of metal atoms confined in molecular dimensions and the special discrete energy levels, metal NCs exhibit dramatically different optical, electronical and chemical properties, including strong photoluminescence, excellent photostability, good biocompatibility, and sub-nanometer size. Such intriguing properties make metal NCs an ideal nanomaterial for various applications including, bioimaging, environmental monitoring, industrial catalysis, and electronic devices. As a result, MNCs were widely explored in the fields of catalysis [14], solar cells [15], biosensors [16], and detection of biomolecules [17]. Recently, significant efforts have been devoted on the exploration of NCs in cancer imaging and therapy owing to its good stability, facile synthesis, excellent biocompatibility, ease of conjugation. Importantly, nanoparticles (NPs) with larger hydrodynamic diameters tend to accumulate more in the reticuloendothelial system (RES) comprising liver and spleen and simply escape from the accumulation in kidneys. This results in serious side organ damages. On contrary, NCs (<10 nm) tend to accumulate in kidney and thereby undergoing renal clearance [18,19]. Therefore, intensive studies are focused on the synthesis of MNCs. After several efforts, Au NCs exhibit as a promising candidate owing to its unique properties and successfully applied to bioimaging of cancer cells both in vitro and in vivo systems [20]. However, some studies reported that Au NCs induce cytotoxicity which might be originating from the capping agents/surface functionalities such as thiol agents [21]. Thereafter, several kind of MNCs such as Ag, Pt, Zn, Mo, and some Cu based MNCs, were reported and assayed their cell labeling properties. To the best of our knowledge, bioimaging capabilities of Pd NCs has not been demonstrated yet.

**Scheme 1:**
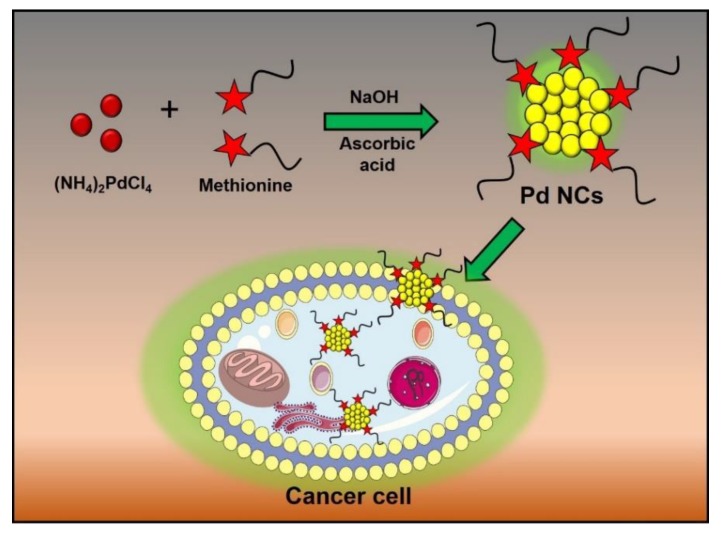
Schematic representation of the synthesis and biolabeling of cancer cells by using Pd NCs.

In this study, Pd nanoclusters with ultra-smaller sizes were synthesized and characterized using various spectroscopic and microscopic techniques. As synthesized Pd NCs exhibit bright green fluorescence emission (500 nm) upon photoexcitation using blue light excitation (420 nm). Furthermore, Pd NCs also showed lower cytotoxicity even at prolonged incubation times. Finally, the bioimaging capabilities of as synthesized Pd NCs in human cervical cancer cells (HeLa) cancer cells were demonstrated. The schematic illustration for the synthesis of biocompatible Pd NCs for the visualization of cancer cells were shown in Scheme 1. 

## 2. Materials and Methods 

### 2.1. Materials

DL-methionine (C_5_H_11_NO_2_S, 99.9%) and Ammonium tetrachloropalladate (II) ((NH_4_)_2_PdCl_4_, 98%) ascorbic acid (C_6_H_8_O_6_, 99.9%), and sodium hydroxide (NaOH) were purchased from Sigma Aldrich. HeLa cells were purchased from Food Industry Research and Development Institute (Hsinchu City, Taiwan). All reagents were used as received without further purification.

### 2.2. Synthesis of Pd Nanoclusters

Pd NCs were synthesized by using a previously reported method [22]. Briefly, all the glassware was cleaned with aquaregia (3:1 conc. HCl/HNO_3_ v/v) followed by a through wash with ultra-pure deionized water. In a typical experiment, 0.1 M DL-Methionine (24 mL) aqueous solution was mixed with 2.5 mM of (NH_4_)_2_PdCl_4_ (12 mL) and 0.6 M of NaOH (3.6 mL) aqueous solution and the contents were stirred for 30 min at room temperature. Subsequently, 0.14 M of L-ascorbic acid (9 mL) was added to the above mixture at 60 °C and kept for 5.5 h. After 5.5 h, the solution was then centrifuged at 8000 rpm for 10 min to remove the larger particles and dialyzed with water using dialysis membrane (1000 Da) to remove the unreacted ligands. Finally, Pd NCs were stored at 4–6 °C in dark.

### 2.3. Characterization

High resolution Transmission Electron Microscopy (HR-TEM) images were obtained on JEOL JEM-2100 (electron microscope operating at 200 kV, Jeol JEM-2100F, Tokyo, Japan). Particle sizes were obtained from DLS (Nanotrac Wave) and FT-IR data recorded on Bruker Tensor27 FT-IR (DTGS detector and KBR beam splitter, Bruker, Vertex 80v and Tensor 27, Billerica, MA, USA). UV-visible absorption spectra obtained from JASCO V-570 UV-vis spectrophotometer (JASCO, Tokyo, Japan) and Photoluminescence emission spectra was recorded on fluorescence spectrometer (FLS920, equipped with a 450 W broadband Xe lamp, Edinburgh). In vitro cellular viabilities measured by using Infinite 200 (TECAN, Männedorf, Switzerland). Fluorescence images of cancer cells were recorded using a confocal laser scanning microscope (CLSM; Leica, TCS SP5X, LSM 700, Zeiss, Jena, Germany) equipped with an InGaN semiconductor laser (405 nm), an Ar laser (488 nm), and a He–Ne laser (533 nm), respectively. 

### 2.4. Cell Culture

Cancer cells such as human cervical cancer cells (HeLa) were grown in Dulbecco’s modified Eagle medium (DMEM, Gibco, Waltham, MA, USA) supplemented with 10% fetal bovine serum (FBS) (Invitrogen, Carlsbad, CA, USA), 2 × 10^−3^ M l-glutamine, 100 μg·mL^−1^ penicillin and 100 U·mL^−1^ streptomycin, placed and grown in a humidified incubator at 37 °C (95% humidity, 5% CO_2_).

### 2.5. MTT Cytotoxicity

HeLa cells were cultured and incubated overnight at 37 °C so that the cells are attached onto the surface of cell cultured plate. To this, various concentrations of Pd NCs and DL-Methionine were added into the 12-well plate containing 1 mL of HeLa cell solution (1 × 10^4^ cells/mL) and incubated for 24 and 48 h in the dark, respectively. Then 50 μL of MTT (3-(4,5-dimethylthiazol-2-yl)-2,5-diphenyl tetrazolium bromide) aqueous solution (0.5 mg·mL^−1^) was added to each well contained cells and further incubated for 4 h. After 4 h, the supernatant was removed carefully and 1 mL of dimethyl sulfoxide (DMSO) was added into each well in order to lyse the cell membrane. Finally, solution in each well was centrifuged at 10,000 rpm for 10 min and the resulting supernatant was collected and the absorbance of each well was measured at 570 nm using ELISA instrument. 

### 2.6. In vitro Bioimaging of Cancer Cells and Cellular Uptake Studies

For bioimaging studies, HeLa cells (2.0 × 10^5^ cells per well) were cultured in a 12-well plate with cover slips and incubated for overnight at 37 °C so that the cells are attached onto the surface of cell cultured plate. To this, 50 and 100 μg/mL of Pd NCs and DL-Methionine were added to the HeLa cells and incubated for 12 h. The cells were then fixed using paraformaldehyde solution (4%) in PBS for 5 min, and then washed with PBST (5% Tween-20 in phosphate buffer solution) solution for three times. Finally, the cells were stained with 4′,6-diamidino-2-phenylindole (DAPI; 1 ng/mL PBS) for 30 min and examined under confocal laser scanning microscope (CLSM; Leica, TCS SP5X, LSM 700, Zeiss, Jena, Germany) equipped with an InGaN semiconductor laser (405 nm), an Ar laser (488 nm), and a He–Ne laser (533 nm), respectively. The fluorescence emission from DAPI and Pd NCs were collected from blue and green channels respectively. For time dependent cellular uptake studies, the same procedure was adopted except Pd NCs concentration at a fixed concentration of 100 μg·mL^−1^ was added and the incubation times were varied from 4, 8, and 12 h.

## 3. Results

### 3.1. Synthesis and Characterization of Pd NCs

**Scheme 2:**
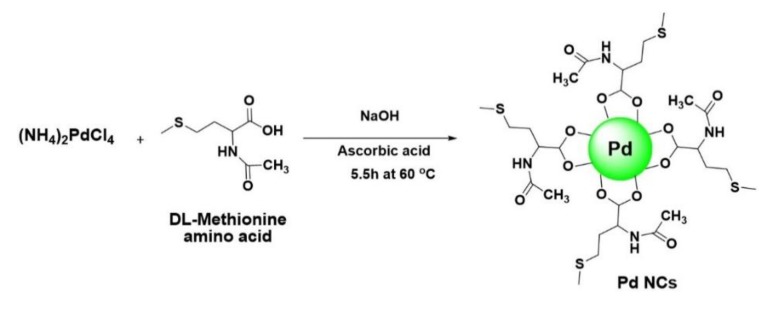
Schematic representation for the synthesis of DL-Methionine capped palladium nanoclusters (Pd NCs).

The water soluble Pd NCs were synthesized using a literature reported procedure in which DL-Methionine and L-ascorbic acid was used as a capping agent and reducing agent [22]. In the previously reported method, there are various factors, such as temperature, concentrations of the reactants (DL methionine, L-ascorbic acid) etc. can influence the formation of Pd NCs. In the current work, we have adopted the optimized experimental conditions from the literature reported method for the synthesis of water soluble Pd NCs (Scheme 2). 

**Figure 1:**
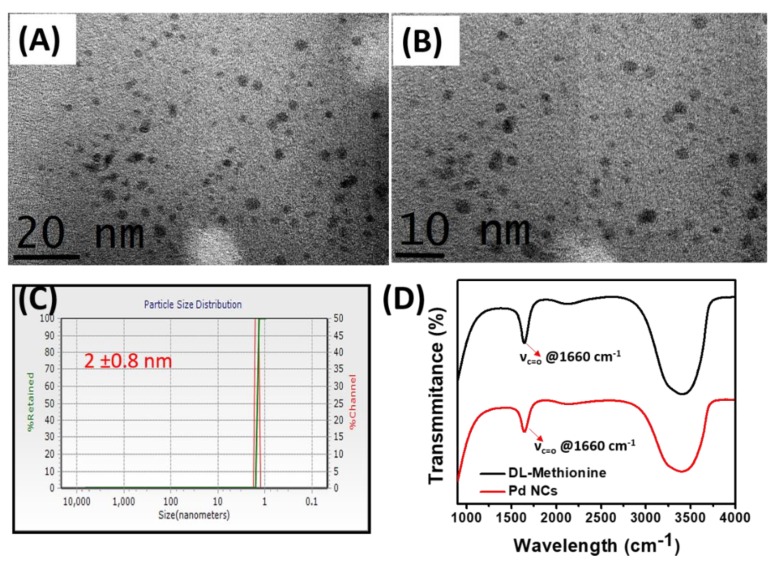
Characterization of Pd NCs. (**A**,**B**) represents the high resolution transmission electron microscopy (HR-TEM) images of Pd NCs. (**C**) Dynamic light scattering (DLS) size distribution of Pd NCs. (**D**) FT-IR spectra of Pd NCs and DL-Methionine.

The obtained Pd NCs were systematically subjected to various spectroscopic and microscopic characterization. First, to identify the successful formation of Pd NCs, high resolution transmission electron microscopy (HR-TEM) analysis was employed. As shown in Figure 1A,B, the low and high magnification TEM images clearly reveal the morphology and uniform distribution Pd NCs with an average diameter 2.0 ± 0.8 nm. In addition, the hydrodynamic diameters and its distribution were also confirmed by dynamic light scattering (DLS) analysis (Figure 1C). The zeta-potential of DL-Methionine capped Pd NCs is −2.4 ± 0.5 mV respectively, indicating that they are slightly negatively charged (data not shown). The presence of capping agent, DL-Methionine on the surface of Pd NCs was also confirmed by using FT-IR spectroscopy (Figure 1D). The FT-IR characteristic peaks at 1660 cm^−1^ correspond to the C=O stretching which was clearly observed in DL-Methionine as well. This result reaffirms the successful capping of DL-Methionine on Pd NCs. 

### 3.2. Optical Properties of Pd NCs

**Figure 2:**
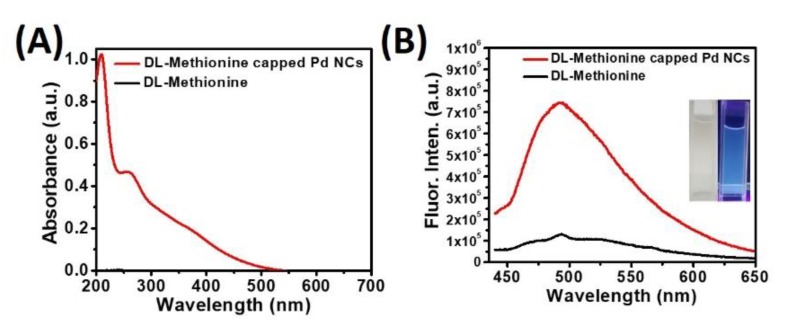
Optical and fluorescence properties of Pd NCs. (**A**) UV-vis absorbance spectra and (**B**) fluorescence emission spectra of Pd NCs and DL-Methionine at a photoexcitation wavelength of 420 nm (inset represents the bluish green fluorescence images of Pd NCs under UV lamp, 365 nm).

The optical properties of as synthesized water soluble Pd NCs were examined using various spectroscopic techniques. Figure 2A represents the extinction spectrum of Pd NCs, in which a very significant optical absorption was observed in the region between 200–500 nm, with a characteristic peak centered at a wavelength of 265 nm and an absorption hump centered at 370 nm. In contrast, there was no noticeable absorption was observed for the surface capping agent, DL-Methionine alone. It is very well known that nanoclusters exhibit appreciable photoluminescence properties owing to its formation of discrete energy levels [23]. To this end, we have examined the fluorescence properties of water soluble Pd NCs. As shown in Figure 2B, a very strong green fluorescence emission was observed with a characteristic peak centered at 500 nm using 420 nm excitation wavelength. The calculated strokes shift is around 80 nm, which is in good agreement with the literature report [22]. On the other hand, DL-Methionine alone was used as a control and there was no significant fluorescence emission observed under the same excitation wavelength conditions. 

The regular photograph under room light and fluorescence image of aqueous solution of Pd NCs were show in the inset in Figure 2B. Upon 365 nm light excitation (using a hand-held UV lamp), a bright bluish green fluorescence was observed from Pd NCs. The fluorescence quantum yield of DL-methionine capped Pd NCs is ~5.47% respectively, which is slightly higher than the literature reported methionine-stabilized fluorescent Au NCs and other metal nanoclusters [22].

**Figure 3:**
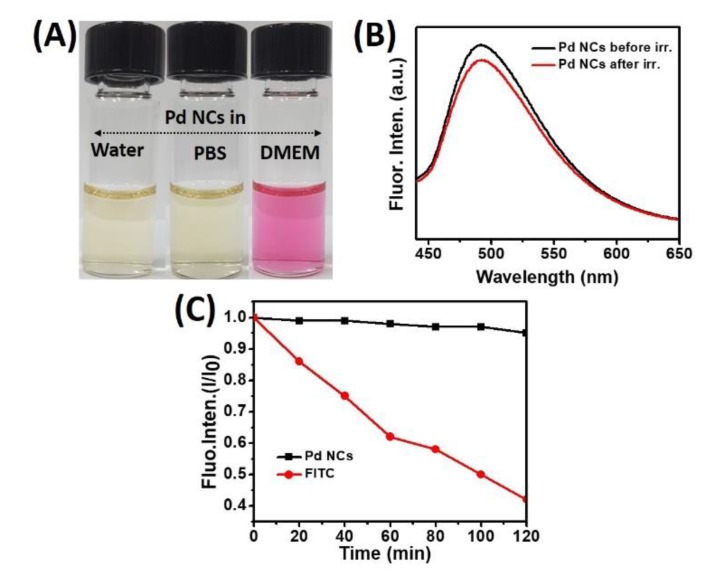
Colloidal and photostability properties of Pd NCs. (**A**) colloidal stability of Pd NCs in various media: water, PBS and DMEM. (**B**) Fluorescence emission spectra of Pd NCs before and after photoillumination at a photoexcitation wavelength of 420 nm. (**C**) Photostabilities of Pd NCs upon comparison with fluorescein isothiocyanate (FITC) dye under exposure of 100 W Hg lamp (output power intensity: 200 mW/cm^2^ at 365 nm wavelength) for 2 h.

Furthermore, to utilize these as synthesized water soluble Pd NCs as in vitro bioimaging probes, it is mandatory to investigate their colloidal stability in various biological media as well as their photostabilities. The colloidal stability of Pd NCs was examined in three different media: Water, phosphate buffered saline (pH ~7.4) and DMEM cell culture media (containing 10% fetal bovine serum) (Figures S1 and S2). It showed excellent stability without any serious aggregation (Figure 3A). 

It is well known that bioimaging probes based on molecular dyes undergo severe photobleaching problems, which can severely limit the overall efficacies. The photostabilities of Pd NCs were investigated before and after irradiation using a high pressure 100 W Hg lamp for 30 min. As shown in Figure 3B, it is very clear that Pd NCs did not induce any noticeable fluorescence decay, which ensures the excellent photostability nature of Pd NCs. Furthermore, we also compared the photostability of DL-Methionine capped Pd NCs to that of conventional molecular dye, fluorescein isothiocyanate (FITC) upon exposure to 100 W Hg lamp for 2 h. As shown in Figure 3C, FITC exhibited significant degradation and whereas Pd NCs were fairly stable until 2 h. Previously, the effects of pH and ionic strength on the stability of the NCs were investigated. Pd NCs showed stable fluorescence over a pH range of 6 to 8, and weak fluorescence at pH = 5 [22]. This could be attributed to the reduction of methionine, which is less stable in acidic environments owing to demethylation. Overall, high water solubility, good photostability, and strong fluorescence properties can set a benchmark for Pd NCs to be able to serve as an in vitro bioimaging probes for efficient visualization of cancer cells.

### 3.3. In Vitro Toxicity of Pd NCs

**Figure 4:**
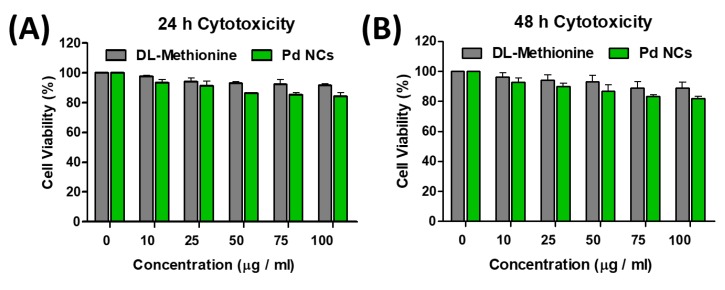
In vitro cytotoxicity studies in human cervical cancer cells (HeLa) cancer cells. (**A**,**B**) represents the percentage cellular viabilities of DL-Methionine capped Pd NCs internalized HeLa cells at 24 and 48 h incubation times, respectively. The error bars indicate the standard deviation (SD) values obtained from four replicates (n = 4) for each set of concentration respectively.

For any biomedical applications, it is mandatory to investigate the cytotoxicity of nanomaterials prior to their utilization into biological applications. To examine the cytotoxicity of as synthesized Pd NCs, MTT (3-(4,5-dimethylthiazolyl-2)-2,5-diphenyltetrazolium bromide) cell viability assay was performed in HeLa cells. The cellular viabilities in Figure 4A,B clearly show that Pd NCs induce dose-dependent cytotoxicity behaviors. It is very clear that the half maximal inhibitory concentration (IC_50_) values for Pd NCs are >100 µg·mL^−1^ at both 24 and 48 h incubation times. On the other hand, DL-Methionine also did not induce any noticeable drop in the cellular viabilities (see Figure 4A,B). These results clearly indicate that water soluble and highly fluorescent Pd NCs are non-toxic to HeLa cancer cells even at higher dosages under prolonged incubation times. As expected, the capping agent DL-Methionine also did not show any prominent cytotoxic effects over the prolonged incubation time. This could be mainly attributed to the fact that DL-Methionine is an essential amino acid in humans and it also responsible for the angiogenesis and growth of new blood vessels [24]. From the data above, one can see that these Pd NCs are non-toxic, biocompatible, and highly water dispersible and therefore, these are promising candidates to be used in various biomedical applications. 

### 3.4. In Vitro Bioimaging of Pd NCs Internalized HeLa Cells

**Figure 5:**
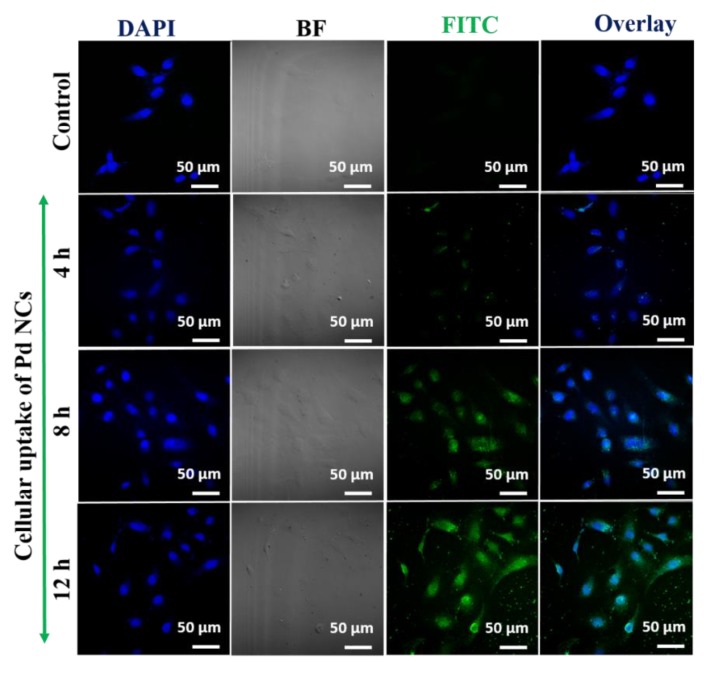
Time dependent cellular uptake of Pd NCs internalized HeLa cells. Green fluorescence originates from the Pd NCs and blue fluorescence corresponds to DAPI which is nucleus staining agent. The concentration of Pd NCs is 100 µg·mL^−1^.

In the recent years, the field of nanomedicine has made a rapid transition from basic to translational research, especially in the areas of biomedical imaging and therapies [25,26]. Owing to its smaller hydrodynamic diameters, nanoclusters can potentially overcome many physiological and biological barriers, which can potentially make them useful for bioimaging to probe the cellular and molecular interactions [9,27]. To investigate the utility as potential intracellular fluorescent markers, HeLa cells were incubated with Pd NCs at different incubation times (4, 8, and 12 h) and monitored their cellular uptake dynamics using a confocal laser scanning optical microscope (CLSM). The CLSM images shows that Pd NCs were readily internalized by the HeLa cells and primarily localized in the cytoplasm region, with a certain fraction entering into the nucleus. This was clearly evident from the green fluorescence originating from the Pd NCs internalized HeLa cells, and gradually enhancing upon increasing the incubation or interaction times (Figure 5). In general, most of the nanomaterials need to internalize across the cell membrane via endocytosis process. However, to facilitate the entry into the nucleus, a small nuclear localization signal (NLS) is required to assist the binding of nanoparticles to the nuclear envelope. The diameter of the nuclear envelope is typically <2 nm. There are also several other barriers which can restrict the entry of nanomaterials into nucleus, such as lysosomal degradation. Nanomaterials have highly active surfaces and surface charge is a significant factor in determining their biological characteristics. Indeed, surface charge has a distinct impact on cellular uptake and cytotoxicity and can vary with the cell type [28,29]. Surface charge can also affect adhesion to cell membranes as well as uptake efficiency [30]. Positively charged nanomaterials can bind strongly to the serum components in the blood via noncovalent interactions with proteins and electrostatic interactions with the cell surface [31]. Furthermore, these positively charged NPs can be easily taken up by nonphagocytic cells and cause more disruption of the membrane integrity and lysosomal and mitochondrial damage than their negatively charged counterparts [32,33,34]. 

**Figure 6:**
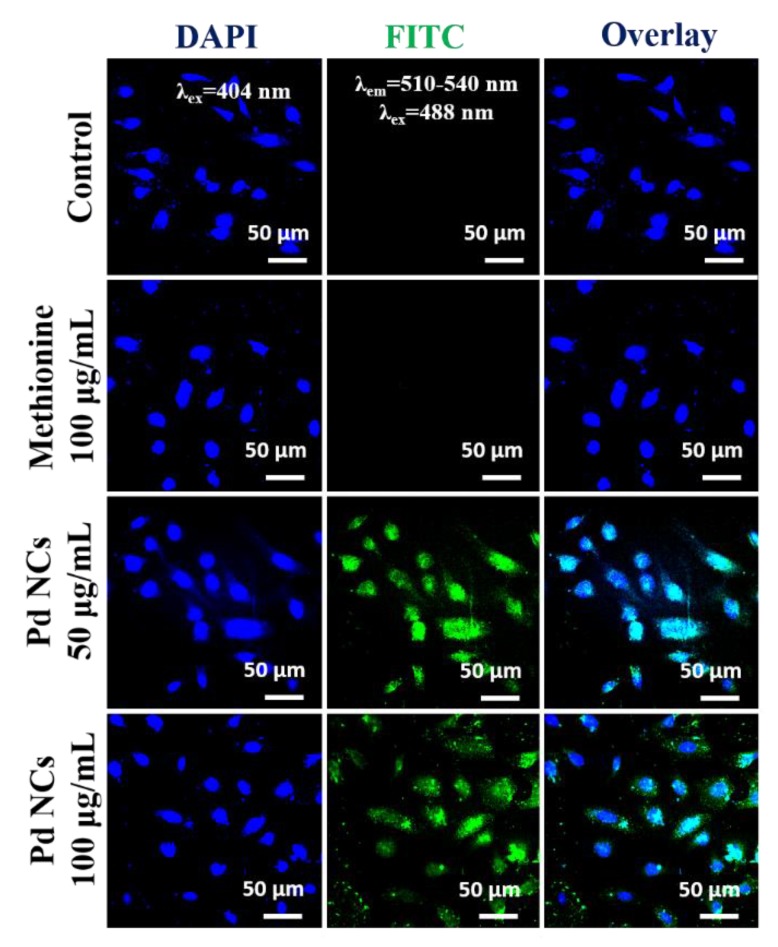
In vitro cell labeling properties of Pd NCs. HeLa cells were treated with various concentrations of Pd NCs and DL-Methionine. Blue fluorescence represents the nucleus which was stained with DAPI and green fluorescence originating from Pd NCs.

**Figure 7:**
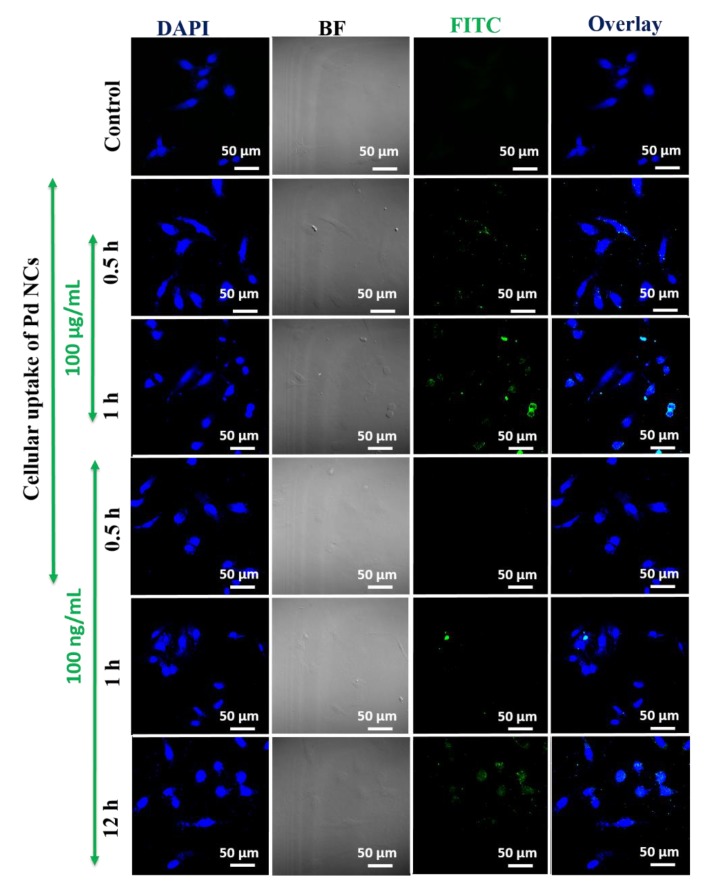
In vitro cell labeling properties of Pd NCs at shorter incubation times (0.5 and 1 h, 100 µg·mL^−1^) and lower doses (100 ng·mL^−1^, 0.5, 1 and 12 h).

We have also evaluated the concentration dependency on the abilities of water soluble Pd NCs as an intracellular biomarker for the detection of cancer cells. As shown in Figure 6, Pd NCs (50 and 100 µg·mL^−1^) internalized HeLa cells exhibits a bright green fluorescence emission and whereas control group and DL-Methionine group did not show any noticeable fluorescence. As shown in the Figure 7, it is very clear that Pd NCs can be internalized by the cancer cells within short incubation times (0.5 h and 1 h of incubation, 100 µg·mL^−1^). However, at lower incubation times, the fluorescence intensities of Pd NCs internalized HeLa cells was lower and hence longer incubation times are recommended. Besides incubation times, concentration of Pd NCs can also affect the HeLa cell labeling efficiency. Therefore, we have carried out bioimaging experiments using ultra low concentration of Pd NCs (100 ng·mL^−1^; 1000 times lower than 100 µg·mL^−1^) for 0.5, 1, and 12 h incubation times. Despite of using lower dosages of Pd NCs, HeLa cells can still be visualized at 12 h incubation time, respectively. Overall, these bioimaging results clearly reveal that with 12 h incubation time, Pd NCs can be effectively uptaken by the HeLa cells without inducing any significant cytotoxicity effects. Therefore, biocompatible and highly fluorescent Pd NCs can be used as an intrinsic fluorescent biomarker to visualize the cancer cells efficiently.

## 4. Discussion

As we discussed earlier, the major problems associated with the conventional organic fluorophores are severe photobleaching problems and poor fluorescence quantum yields. To overcome the above limitations, several methods were employed to synthesize MNCs which are potentially photostable, biocompatible, water soluble, and appreciable fluorescence quantum yields. The comparison of literature reported metal nanoclusters as intracellular fluorescent markers for visualization of cancer cells were summarized in Table 1. 

Among various MNCs family, Au NCs exhibit superior capabilities to visualize the cancer cells/solid tumors efficiently [20]. In contrast to gold nanoparticles, noble metal-based nanoparticles (e.g., platinum (Pt) and palladium (Pd)) are not commonly used as drug delivery vehicles in the biomedical applications because they tend to show high cytotoxicity, not cost-effective, and exhibit limited localized surface plasmon resonance (LSPR) properties in the UV region [35,36]. Previously, the photothermal conversion efficiencies, gene/drug loading and release, cytotoxicity, and combination of cancer treatments through manufactured porous Au, Pt, and Pd nanoplates were reported [37]. Recently, several Pd based nanomaterials gained significant attention in cancer phototherapies owing to its superior plasmonic properties [38]. However, to the best of our knowledge, utilization of Pd NCs as an intracellular fluorescent marker has not been investigated. Therefore, in the current study, we have successfully synthesized water soluble, biocompatible, and highly fluorescent Pd NCs, which can emit green fluorescence emission under blue light (420 nm) excitation. It is well known that particles sizes in quantum range exhibits some distinguishable properties mainly due to the interstitial space between the energy levels, which is inversely proportional to the size of particles so called quantum confinement effects [39].

In addition to the exciting optical properties, Pd NCs also exhibits excellent photostabilities. To the best of our knowledge, utilization of Pd NCs for nanomedicine such as bioimaging of cancer cells and monitoring the cellular uptake and investigating its cytotoxicity has not been demonstrated. Other than size and shape of nanomaterials, surface functionalities also play a prominent role in dictating the short-term cytotoxicities [40]. Keeping that in mind, we have investigated the percent cellular viabilities of DL-Methionine alone in HeLa cells and it did not induce any noticeable cytotoxicity. It is known that amino acids are biocompatible and as a result an amino acid such as DL-Methionine prevents the NCs from agglomeration and also diminishing the overall toxicity of MNCs.

**Figure 8:**
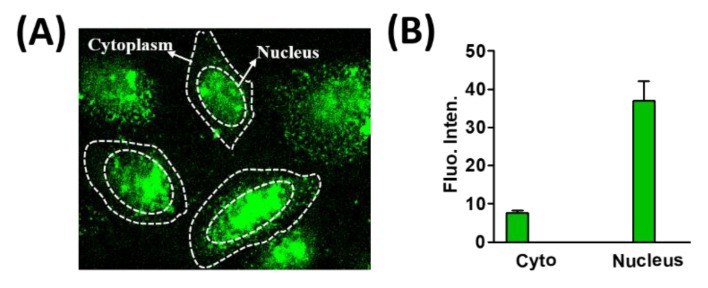
Translocation of Pd NCs inside the HeLa cell nucleus. (**A**,**B**) represent the confocal laser scanning optical microscope (CLSM) image and mean fluorescence intensities from nucleus and cytoplasm respectively.

As a result, we did not observe any noticeable cytotoxic effects mediating from Pd NCs and DL-methionine internalized HeLa cells even at prolonged time of incubations such as 24 and 48 h, respectively. Furthermore, we also observed the translocation of Pd NCs into the HeLa cell nucleus. Notably, most of the green fluorescence from the Pd NCs was originating from the cell nucleus (see Figure 8A). This could be presumably due to the ability of Pd NCs accumulate in the nucleus via passive targeting [41]. As shown in Figure 8B, the mean fluorescence intensities from Pd NCs internalized HeLa cells from the nucleus is 5 fold higher than the intensities observed in the cytoplasm. The cellular uptake capabilities of Pd NCs with specific molecular targeting is currently ongoing. Overall, ultra-small size and biocompatible Pd NCs were able to accumulate more in nucleus via passive targeting. These nucleus translocation properties of Pd NCs without any NLS peptide on the surface can certainly draw significant attention from the scientific community for future potential biomedical applications. 

## 5. Conclusions

In summary, we have successfully fabricated water soluble, biocompatible and fluorescent palladium nanoclusters (Pd NCs) by using L-Ascorbic acid reducing agent and DL-Methionine as a stabilizer/capping agent with an average hydrodynamic diameter of ~2.0 nm. These nanoclusters showed excellent photophysical properties with the characteristic emission peak centered at 500 nm under 420 nm photoexcitation wavelength. Protection of essential amino acid DL-Methionine can serve as a biocompatible coating on the surface of Pd NCs. In vitro cytotoxicity studies in HeLa cells reveal that Pd NCs exhibited good biocompatibility with an IC_50_ value of >100 µg·mL^−1^ and also showed excellent co-localization and distribution throughout the cytoplasm region with a significant fraction translocating into cell nucleus. To the best of our knowledge, this is the first demonstration of Pd NCs serving as intrinsic intracellular fluorescent markers for visualization of cancer cells. Overall, water soluble, biocompatible and highly fluorescent Pd NCs has utmost clinical relevance in the biomedical clinics to be utilized as fluorescent sprays to visualize tiny tumor nodules and also in the image-guided surgeries. 

## Figures and Tables

**Table 1:** Comparison of literature reported metal nanoclusters as intracellular fluorescent markers for visualization of cancer cells.

S. No	Metal Cluster	Fluorescence Emission (λ_em_)	Cancer Cells	Reference
1	Au NCs	650 nm	HeLa cells	[42]
2	Ag NCs	450 nm, 570 nm, 720 nm	A549 cells	[43]
3	Pt NCs	460 nm	HepG2, HeLa, HCT116, A549 cells	[44]
4	Cu NCs	450 nm	OAW42 cell	[45]
5	IrO_2_ NCs	750 nm	HeLa, HepG2 cell	[46]
6	Au/Ce NCs	570 nm	HeLa, HepG2, L02 cells	[47]
7	CuAu NCs	610 nm	HeLa cells	[48]
8	Zn NCs	640 nm	HeLa cells	[49]
9	Mo NCs	494 nm	HaCaT, A549, RPTEC cells	[50]
10	Pd NCs	500 nm	HeLa cells	Present work

## Supplementary Materials

The following are available online at https://www.mdpi.com/2306-5354/7/1/20/s1, Figure S1: Absorption spectra of Pd NCs in various biological media medium, Figure S2: Stability of Pd NCs in DMEM cell culture medium with and without 10% FBS.
