# Supplementary material for: Preparation, Cytotoxicity, and In Vitro Bioimaging of Water Soluble and Highly Fluorescent Palladium Nanoclusters
Source: Bioengineering (Basel). 2020 Feb 21;7(1):20. doi: 10.3390/bioengineering7010020 (PMC7175340; doi:10.3390/bioengineering7010020)
Supplement: Supplementary file 1 [file bioengineering-07-00020-s001.pdf]

bioengineering

Supporting information

Preparation, Cytotoxicity and *In vitro* Bioimaging of Water Soluble and Highly Fluorescent Palladium Nanoclusters

Suresh Thangudu[1], Poliraju Kalluru[2] and Raviraj Vankayala[3*]

[1] Department of Chemistry, National Tsing Hua University, Hsinchu, Taiwan.
[2] Department of Chemistry, University of Calgary, Alberta, Canada.
[3] Department of Bioscience and Bioengineering, Indian Institute of Technology Jodhpur, Jodhpur, Rajasthan, India.
*Correspondence: rvankayala@iitj.ac.in

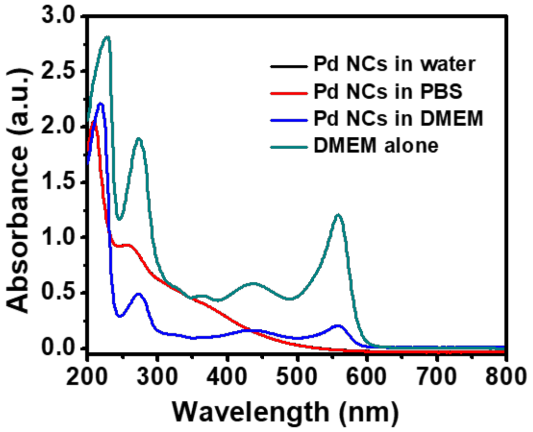

Figure S1. Absorption spectra of Pd NCs in various biological media medium.

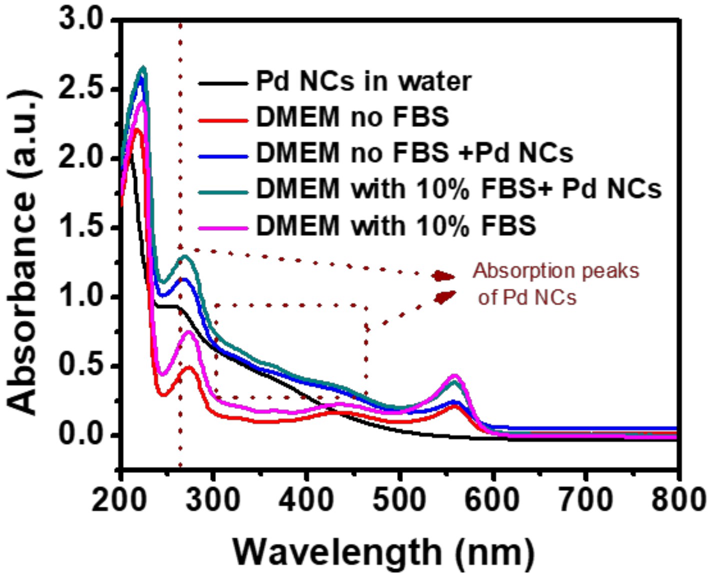

Figure S2. Stability of Pd NCs in DMEM cell culture medium with and without 10% FBS.
